# Development and validation of risk prediction nomograms for acute respiratory failure in elderly patients with hip fracture

**DOI:** 10.1186/s13018-023-04395-z

**Published:** 2023-11-25

**Authors:** Yue Li, Bo Dong

**Affiliations:** Pain ward of Rehabilitation Department, Honghui Hospital, Xi’an Jiaotong University, No. 555 Youyi East Road, Beilin District, Xi’an, 710054 Shaanxi Province China

**Keywords:** Hip fracture, Respiratory failure, Nomogram, MIMIC database

## Abstract

**Background:**

Hip fractures in the elderly often lead to acute respiratory failure, but there is currently no tool to assess the prognosis of such patients. This study aims to develop a risk prediction model for respiratory failure in these patients.

**Methods:**

A retrospective cross-sectional study was conducted using the Medical Information Mart for Intensive Care (MIMIC)-IV database, incorporating data from 3,266 patients with hip fractures aged over 55 years from 2008 to 2019. Data included demographic information, laboratory indicators, comorbidities, and treatment methods. Patients were divided into a training group (70%) and a validation group (30%). Least Absolute Shrinkage and Selection Operator (LASSO) regression was applied to select prognostic predictors, and a visualized nomogram model was constructed using multivariate logistic regression analysis. Model performance and clinical applicability were assessed. Statistical analyses were done using R4.2.2, with *P* < 0.05 deemed significant.

**Results:**

Seven key factors, including age, height, albumin, chloride, pneumonia, acute kidney injury (AKI), and heparin use, were associated with respiratory failure risk. The model demonstrated good performance with area under the curve (AUC) values of 0.77 and 0.73 in the training and validation sets, respectively. The calibration curve showed good agreement, and decision curve analysis (DCA) indicated the model's clinical benefit.

**Conclusions:**

This risk prediction model can effectively predict respiratory failure in hip fracture patients, assisting clinicians in identifying high-risk individuals and providing evidence-based references for treatment strategies.

## Introduction

Hip fracture is one of the most common traumatic fracture types in the elderly [1]. With the rapid aging of the population, hip fracture in the elderly has been paid increasing attention in the field of health care [2, 3]. The prevalence of hip fractures in the elderly population is so widespread that dedicated orthogeriatricians are even employed for the care of patients with hip fractures [4]. The prevalence of hip fractures exhibits geographical variations. Globally, it is projected that approximately 18% of women and 6% of men will experience a hip fracture. Consequently, worldwide hip fractures are anticipated to rise from 1.26 million cases in 1990 to an alarming 4.5 million by 2050 [5]. Symptoms usually include severe pain, swelling, bruising, abnormal appearance of thighs (such as shortening or eversion) [6]. Common surgical treatment methods include hip joint replacement, intramedullary nail fixation, and other techniques [7, 8].

In addition to the direct impact of the fracture, these patients may also suffer from many serious complications, such as acute respiratory failure, pulmonary embolism, pneumonia, deep vein thrombosis, urinary tract infections, and muscle loss, to name a few [9–11]. Acute respiratory failure not only affects the recovery of patients, but also further leads to declined quality of life, and even becomes life-threatening [12, 13]. Patients with hip fractures often need to stay in bed for long periods of time during rehabilitation, which may increase the risk of lung infection and pulmonary embolism, leading to acute respiratory failure [14, 15]. Furthermore, older people tend to have other chronic diseases, such as heart and lung diseases and diabetes, which may also increase the risk of acute respiratory failure [16, 17]. Therefore, it is very important to evaluate the risk of acute respiratory failure in elderly patients with hip fracture and carry out early intervention. Currently, there is no specific tool or model to assess and predict the risk of acute respiratory failure in these patients.

In this study, clinical data were collected from elderly patients with hip fractures. The Least Absolute Shrinkage and Selection Operator (LASSO) regression analysis was employed to identify potential predictors. A predictive model was established using logistic regression and visualized as a nomogram. The performance of this model was evaluated in both the training and validation sets to assess its robustness in predicting the occurrence of acute respiratory failure in these patients. The study was documented in line with the Transparent Reporting of a multivariable prediction model for Individual Prognosis Or Diagnosis (TRIPOD) report checklist.

## Methods

### Data source

**Fig. 1:**
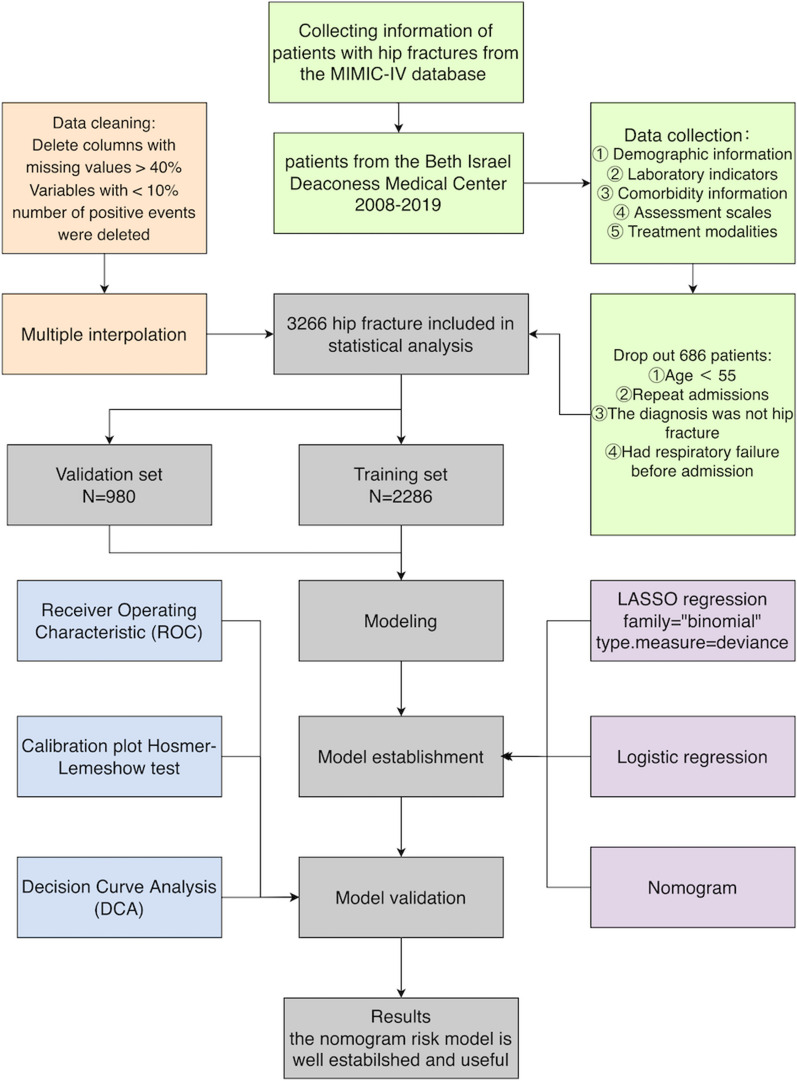
Flow diagram of study design

Data for this retrospective cross-sectional study were obtained from the Medical Information Mart for Intensive Care (MIMIC)-IV database [18]. It is maintained by the Computational Physiology Laboratory at Massachusetts Institute of Technology, encompassing clinical information of 58,000 patients who were admitted to the ICU of Beth Israel Deaconess Medical Center (Boston, Massachusetts, USA) between 2008 and 2019. Enrolled patients were de-identified under the Health Insurance Portability and Accountability Act (HIPAA). A team member (Yue Li) had access to the database and was responsible for data extraction (CITE Number: 55377177). The flow chart of this study was as follows (Fig. 1).

### Study population

Patients with hip fractures in the MIMIC-IV database were included in the study according to the International Classification of Diseases, 9th and 10th editions (ICD-9, ICD10). The ICD codes used are shown in Table 1. Data from 3266 patients with hip fractures were incorporated from the MIMIC-IV database, spanning January 2008 to December 2019. The collection of patient information and creation of the research resource were reviewed by the Institutional Review Board at the Beth Israel Deaconess Medical Center, who granted a waiver of informed consent and approved the data sharing initiative.

**Table 1:** ICD-9 and ICD-10 procedure codes for hip fractures

Codes	Principal diagnoses
820	Fracture of neck of femur
821	Fracture of other and unspecified parts of femur
S72	Fracture of femur
S790	Physeal fracture of upper end of femur
M9666	Fracture of femur following insertion of orthopedic implant, joint prosthesis, or bone plate
M8475	Atypical femoral fracture
M8465	Pathological fracture in other disease, pelvis, and femur
M8455	Pathological fracture in neoplastic disease, pelvis, and femur
M8445	Pathological fracture, femur, and pelvis
M8435	Stress fracture, pelvis, and femur
M8005	Age-related osteoporosis with current pathological fracture, femur
M8085	Other osteoporosis with current pathological fracture, femur
73,314	Pathological fracture of neck of femur
73,396	Stress fracture of femoral neck

The inclusion criteria were as follows: ① patients aged > 55 years; ② patients diagnosed with hip fracture; ③ patients with respiratory failure that meet the diagnostic criteria for respiratory failure. Respiratory failure was defined as an arterial oxygen partial pressure (PaO_2_) below 60 mmHg, which may or may not be accompanied by an arterial carbon dioxide partial pressure (PaCO_2_) above 50 mmHg [19]. The PaO_2_ and PaCO_2_ values for each patient can be obtained from the MIMIC database.

The exclusion criteria were as follows: ① patients < 55 years old; ② patients admitted repeatedly; ③ patients with respiratory failure before admission; ④ patients who were diagnosed with diseases other than hip fractures and did not have concurrent hip fractures.

### Data extraction

The data of hip fracture patients were extracted by structured query language (SQL) (PostgreSQL 15.3) on PgADmin4 (v7.1) software. The extracted data included: ① demographic information: age, gender, height, weight, blood pressure, body mass index (BMI), race, marriage, and insurance information; ② laboratory indicators: albumin, calcium, creatinine, potassium, alkaline phosphatase, phosphorus, chlorine, blood sodium, blood glucose, total bilirubin, alanine aminotransferase (ALT), aspartate aminotransferase (AST), creatine kinase (CK), C-reactive protein (CRP), D-dimer, prothrombin time (PT), erythrocyte hemoglobin distribution width (RDW); ③ comorbidity information: dementia, obesity, pulmonary embolism, pneumonia, cirrhosis, osteoporosis, acute kidney injury (AKI), chronic obstructive pulmonary disease (COPD), cerebral infarction, sepsis, Parkinson's disease, asthma, myocardial infarction, arrhythmia, heart failure; ④ evaluation scale: Simplified Acute Physiology Score II (SAPSII), Sequential Organ Failure Assessment (SOFA) score; ⑤ treatment methods: heparin, mechanical ventilation, antibiotic treatment.

### Nomogram construction

The subjects were randomly divided into a training group (70%) and a validation group (30%). The baseline data were compared between the two groups. The training set was used to build the predictive model, while the validation set was used to evaluate the performance of the model. Multiple imputation was performed for missing values < 40% [20], and outliers were converted to normal values using the capping method. In the course of this study, we employed LASSO regression, a regularized linear regression, to select prognostic predictors primarily due to its proficiency in handling high-dimensional data [21]. Lasso regression introduces an L1 penalty in the loss function, shrinking some coefficients precisely to zero, thereby excluding them from the model [22]. First, LASSO regression was used to select the best predictive feature variables, and lambda 1se was determined as the corresponding coefficient by tenfold cross-validation. Then, multivariate logistic regression analysis was used to determine the risk factors of respiratory failure in hip fracture, and a visualized nomogram model was constructed.

### Nomogram validation and performance evaluation

The concordance index (C-index) and the area under the receiver operating characteristic (ROC) curve (AUC) were calculated in the training and validation groups to assess the accuracy of the nomogram model. Calibration curves were used to evaluate the calibration capability of the nomogram model. The goodness-of-fit of the logistic regression model was assessed using the Hosmer–Lemeshow test. The clinical efficacy of the nomogram model was assessed using decision curve analysis (DCA).

### Statistical analysis

Kolmogorov–Smirnov (K–S) method was used to test the normality of the measurement data. The measurement data of normal distribution were described by mean ± standard deviation, and non-normal distribution data were described by median and upper and lower quartile. Enumeration data were compared by χ^2^ test and described as the number of cases or percentage. Logistic regression analysis could calculate the odds ratio (OR) and 95% confidence interval (CI), and the model with the minimum AIC was selected as the optimal model. Statistical analysis was performed using R4.2.2 software. The invoked packages included: mice package, glmnet package, caret package, rms package, pROC package, and rmda package. All tests were two-sided, and *P* < 0.05 was considered statistically significant. The study was conducted in accordance with the Declaration of Helsinki (as revised in 2013).

## Results

### Baseline information

According to the inclusion and exclusion criteria, there were 3266 hip fracture patients in the MIMIC-IV database, of whom 2942 patients did not develop respiratory failure and 324 patients developed respiratory failure. The data were randomly split in a 7:3 ratio into a training set (*n* = 2286) and a validation set (*n* = 980). The basic information is shown in Table 2. As shown in Table 2, the patients are mainly females, accounting for 70.15% of the total number (*n* = 2288); the proportion of cardiac dysrhythmia in comorbidity information is the highest, reaching 41.12% (*n* = 1342). In the continuous variables, the blood glucose, PT, and RDW values were relatively high, which may reflect the presence of hyperglycemia, coagulation dysfunction, or uneven distribution of red blood cells in the included patient population. There was no statistically significant difference in all indicators between the two groups (*P* > 0.05), and the baseline data were comparable.

**Table 2:** Baseline characteristics between training set and validation set

Category	Items	Group	Total patient cohort (n = 3266)	training set (n = 2286)	Validation set (n = 980)	*P*
Laboratory examinations	Albumin (median [IQR])		3.80 [3.20, 4.20]	3.80 [3.20, 4.20]	3.80 [3.20, 4.20]	0.79
	Calcium (median [IQR])		8.90 [8.40, 9.30]	8.90 [8.40, 9.30]	8.90 [8.40, 9.40]	0.36
	Creatinine (median [IQR])		0.90 [0.70, 1.20]	0.90 [0.70, 1.20]	0.90 [0.70, 1.20]	0.37
	Potassium (median [IQR])		4.20 [3.90, 4.60]	4.20 [3.90, 4.60]	4.20 [3.90, 4.60]	0.61
	Alkaline_phosphatase (median [IQR])		82.00 [64.00, 109.00]	81.00 [65.00, 109.00]	82.00 [64.00, 106.00]	0.51
	Phosphate (median [IQR])		3.40 [2.90, 3.80]	3.40 [2.90, 3.80]	3.30 [2.90, 3.80]	0.97
	Chloride (median [IQR])		102.00 [100.00, 105.00]	102.00 [100.00, 105.00]	102.00 [100.00, 105.00]	0.31
	Sodium (median [IQR])		139.00 [137.00, 141.00]	139.00 [137.00, 141.00]	139.00 [137.00, 141.00]	0.16
	Blood_glucose (median [IQR])		117.50 [99.00, 144.00]	117.00 [99.00, 142.00]	119.00 [100.00, 147.00]	0.22
	Bilirubin (median [IQR])		0.50 [0.30, 0.70]	0.50 [0.30, 0.70]	0.50 [0.30, 0.70]	0.12
	ALT (median [IQR])		18.00 [13.25, 26.00]	18.00 [14.00, 26.00]	18.00 [13.00, 25.00]	0.29
	AST (median [IQR])		24.00 [20.00, 34.00]	24.00 [19.00, 34.00]	24.00 [20.00, 34.00]	0.73
	PT (median [IQR])		12.30 [11.40, 13.50]	12.30 [11.40, 13.40]	12.40 [11.40, 13.62]	0.19
	RDW (median [IQR])		13.80 [13.20, 14.70]	13.80 [13.20, 14.70]	13.80 [13.10, 14.70]	0.47
Demography	Anchor_age (median [IQR])		80.00 [70.00, 87.00]	80.00 [70.00, 87.00]	81.00 [71.00, 88.00]	0.05
	Weight (median [IQR])		148.00 [123.00, 177.08]	148.07 [124.00, 178.00]	147.00 [121.80, 176.50]	0.35
	Height (median [IQR])		64.00 [61.50, 67.00]	64.00 [61.50, 67.00]	64.00 [61.50, 67.00]	0.22
	BMI (median [IQR])		25.30 [22.02, 29.40]	25.30 [22.10, 29.40]	25.40 [22.00, 29.42]	0.59
	Gender (%)	Female	2288 (70.12)	1597 (69.19)	691 (70.50)	0.74
		Male	978 (29.92)	689 (30.13)	289 (29.53)	
	Insurance (%)	Medicaid	87 (2.74)	56 (2.45)	31 (3.20)	0.49
		Medicare	2098 (64.25)	1469 (64.30)	629 (64.20)	
		None	1081 (33.13)	761 (33.39)	320 (32.76)	
	Marital status (%)	Married	1945 (59.64)	1367 (59.83)	578 (59.07)	0.69
		Unmarried	1321 (40.44)	919 (40.25)	402 (41.04)	
	Race (%)	White	645 (19.76)	439 (19.26)	206 (21.02)	0.25
		Non-white	2621 (80.32)	1847 (80.85)	774 (79.07)	
Comorbidity	Dementia (%)	No	2127 (65.15)	1496 (65.42)	631 (64.45)	0.59
		Yes	1139 (34.93)	790 (34.69)	349 (35.63)	
	Pneumonia (%)	No	2929 (89.72)	2032 (88.96)	897 (91.52)	0.03
		Yes	337 (10.37)	254 (11.15)	83 (8.51)	
	Osteoporosis (%)	No	2374 (72.78)	1666 (72.92)	708 (72.29)	0.74
		Yes	892 (27.37)	620 (27.14)	272 (27.88)	
	AKI (%)	No	2368 (72.56)	1652 (72.38)	716 (73.15)	0.67
		Yes	898 (27.52)	634 (27.77)	264 (26.94)	
	COPD (%)	No	2731 (83.61)	1904 (83.34)	827 (84.47)	0.46
		Yes	535 (16.45)	382 (16.71)	153 (15.61)	
	Sepsis (%)	No	2593 (79.46)	1815 (79.46)	778 (79.42)	1.00
		Yes	673 (20.67)	471 (20.66)	202 (20.63)	
	Diabetes (%)	No	2434 (74.51)	1693 (74.12)	741 (75.66)	0.37
		Yes	832 (25.58)	593 (25.96)	239 (24.44)	
	Myocardial infarction (%)	No	2230 (68.39)	1545 (67.62)	685 (69.95)	0.21
		Yes	1036 (31.75)	741 (32.43)	295 (30.17)	
	Cardiac dysrhythmia (%)	No	1924 (58.97)	1366 (59.88)	558 (56.94)	0.15
		Yes	1342 (41.13)	920 (40.26)	422 (43.16)	
	Heart failure (%)	No	2285 (70.04)	1613 (70.64)	672 (68.62)	0.27
		Yes	981 (30.02)	673 (29.46)	308 (31.45)	
treatment	Heparin (%)	No	1039 (31.81)	717 (31.42)	322 (32.93)	0.42
		Yes	2227 (68.26)	1569 (68.63)	658 (67.12)	
	Antibiotic treatment (%)	No	167 (5.17)	116 (5.15)	51 (5.23)	0.94
		Yes	3099 (94.96)	2170 (94.91)	929 (94.81)	
	Ventilation status (%)	No	2375 (72.75)	1665 (72.83)	710 (72.42)	0.85
		Yes	891 (27.32)	621 (27.21)	270 (27.61)	

### LASSO analysis and logistic regression results

**Fig. 2:**
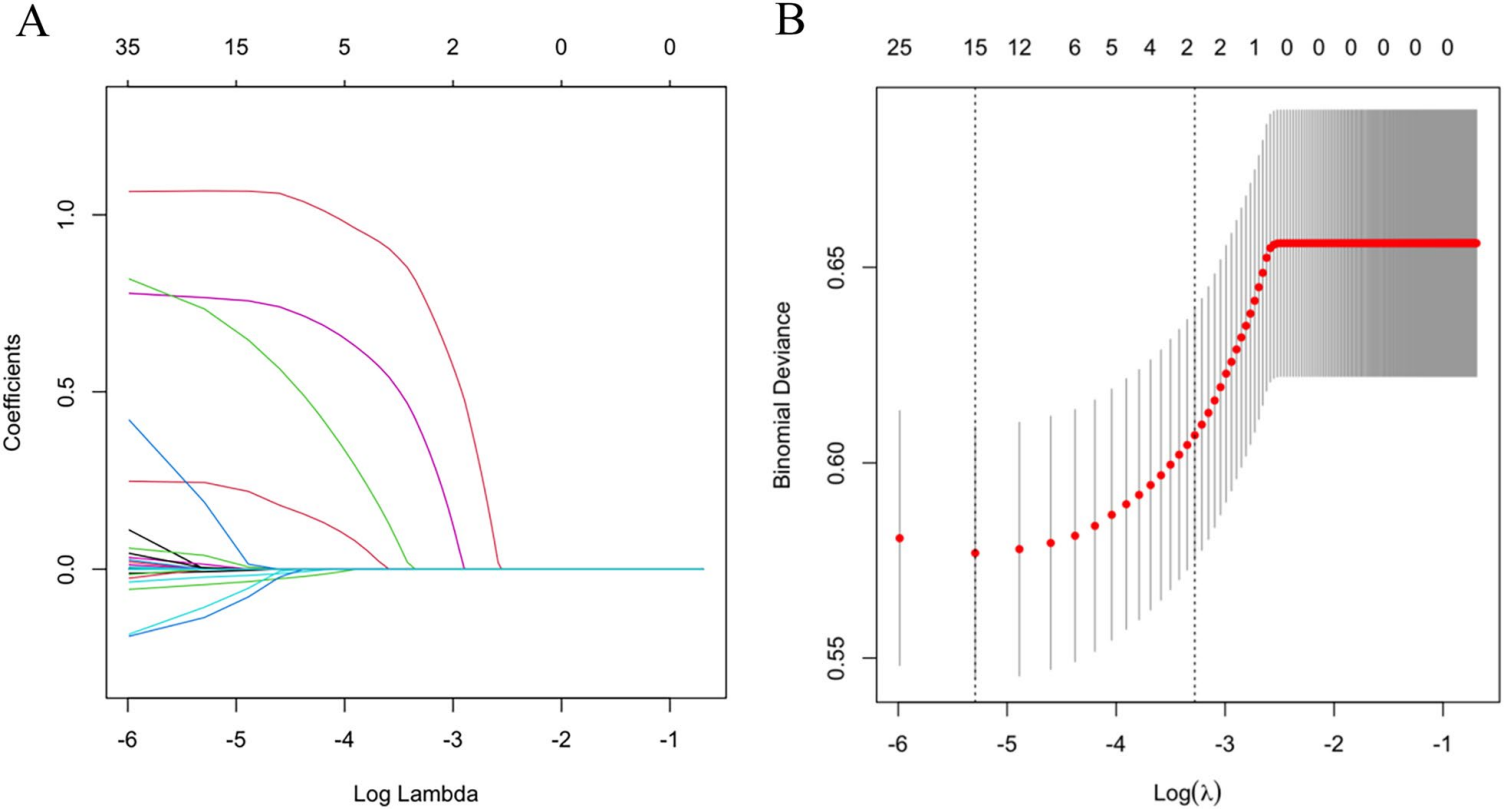
Clinical variables were selected using the LASSO logistic regression model tuning parameter (λ) selection using LASSO penalized logistic regression with tenfold cross-validation. LASSO coefficient profiles of the radiomic features

All 34 variables were included in the LASSO regression to screen for characteristic variables. When the adjustment parameter was lambda. 1se (*λ* = 0.005), the candidate predictors of the initial screening include the following 15 variables (Fig. 2): age, height, albumin, creatinine, chloride, AST, race, pneumonia, AKI, COPD, myocardial infarction, cardiac dysrhythmia, heart failure, heparin, and antibiotic treatment. According to the results of LASSO regression analysis, the 15 candidate predictors were included in the multivariate logistic regression model. Multivariate analysis showed that 7 variables were associated with the risk of respiratory failure in patients with hip fracture (Table 3), including age (OR: 0.98, 95%CI:0.97–0.99), height (OR: 0.94, 95%CI: 0.91–0.97), albumin (OR: 0.80, 95%CI: 0.65–0.99), chloride (OR: 0.97, 95%CI: 0.94–0.99), pneumonia (reference: without pneumonia, OR: 2.18, 95% CI: 1.53–3.12), AKI (reference: without AKI, OR: 2.89, 95% CI: 2.10–4.02), and heparin (reference: without heparin, OR: 2.54, 95%CI: 1.54–4.20).

**Table 3:** Results of multivariate logistic regression

Level	OR	CI	P
Anchor_age	**0.98**	**0.97–0.99**	**0.04**
Height	**0.94**	**0.91–0.97**	**0.00**
Albumin	**0.80**	**0.65–0.99**	**0.04**
Creatinine	1.04	0.91–1.19	0.57
Chloride	**0.97**	**0.94–0.99**	**0.03**
Ast	0.99	0.99–1.00	0.16
Race	0.77	0.55–1.09	0.14
Pneumonia	**2.18**	**1.54–3.12**	**0.00**
Aki	**2.89**	**2.10–4.02**	**0.00**
Copd	1.11	0.78–1.58	0.55
Myocardial_infarction	1.05	0.75–1.47	0.76
Cardiac_dysrhythmia	1.11	0.79–1.54	0.55
Heart_failure	1.29	0.91–1.84	0.15
Heparin	**2.54**	**1.54–4.20**	**0.00**
Antibiotic treatment	1.98	0.71–5.55	0.19

Note: Variables significantly associated with the risk of respiratory failure in patients with hip fractures are highlighted in bold (P < 0.05)

### Nomogram building and model performance evaluation

**Fig. 3:**
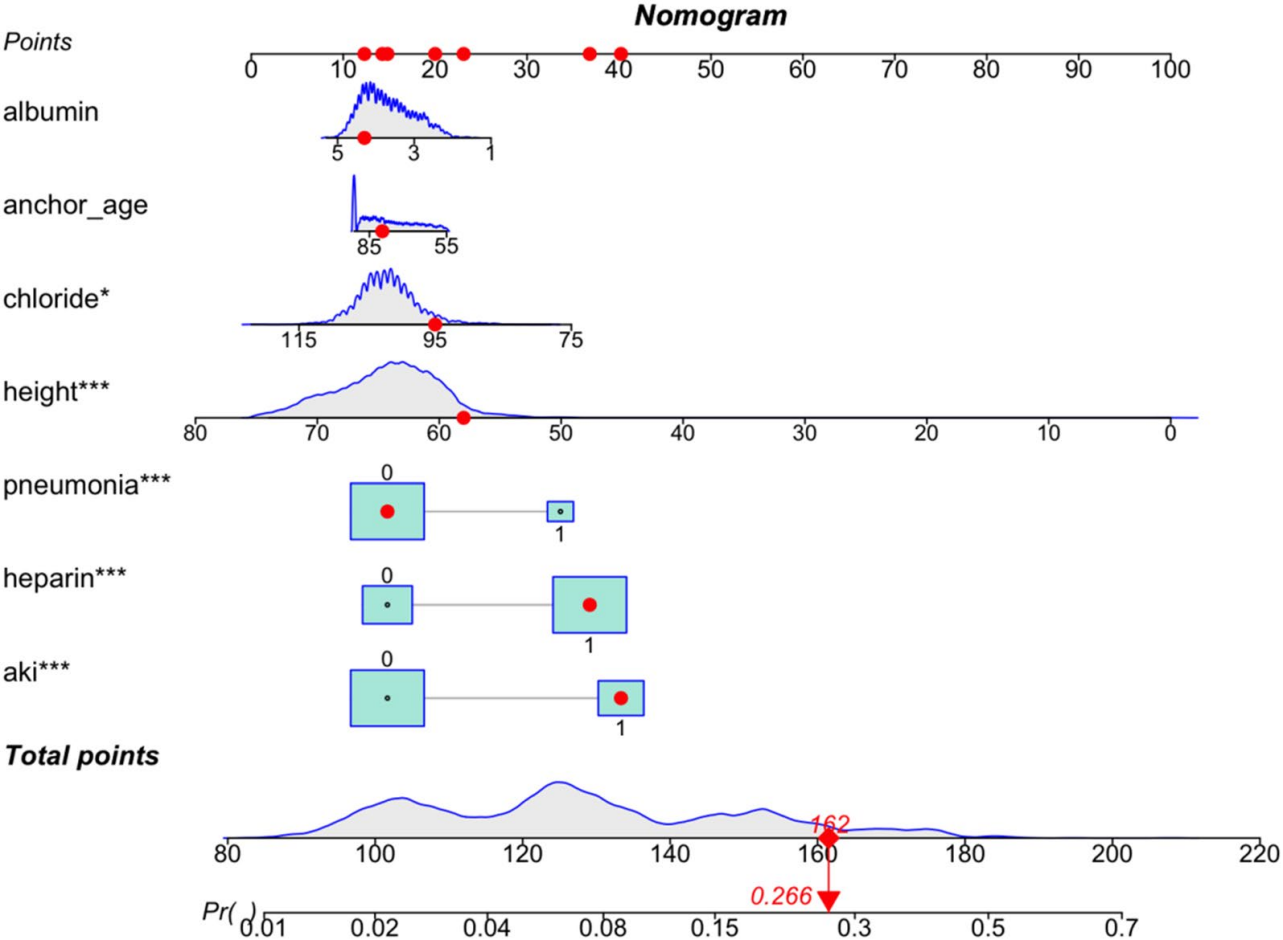
A line chart of the in-hospital mortality rate of patients with femoral fracture complicated by respiratory failure

**Fig. 4:**
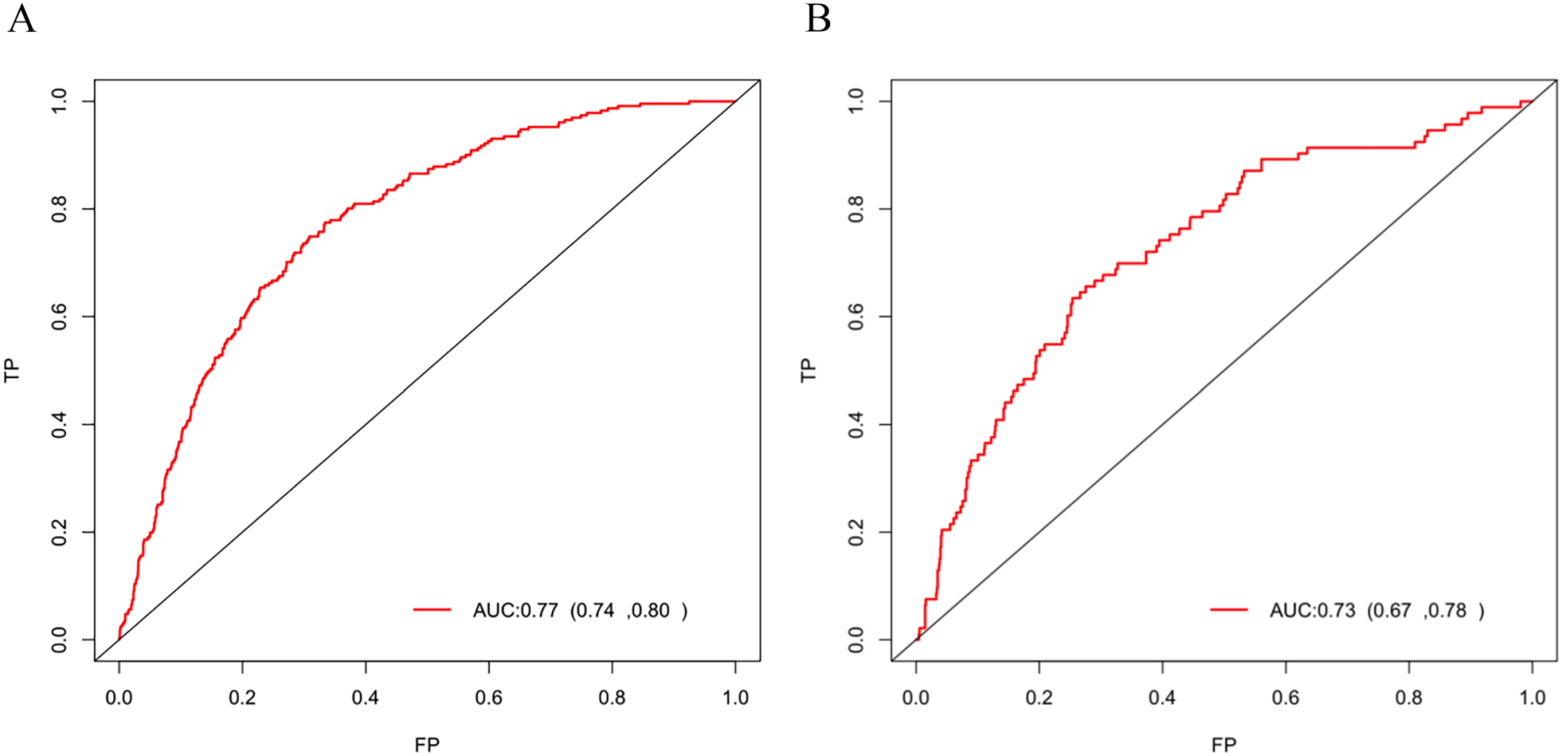
ROC curves. **A** training set; **B** validation set

**Fig. 5:**
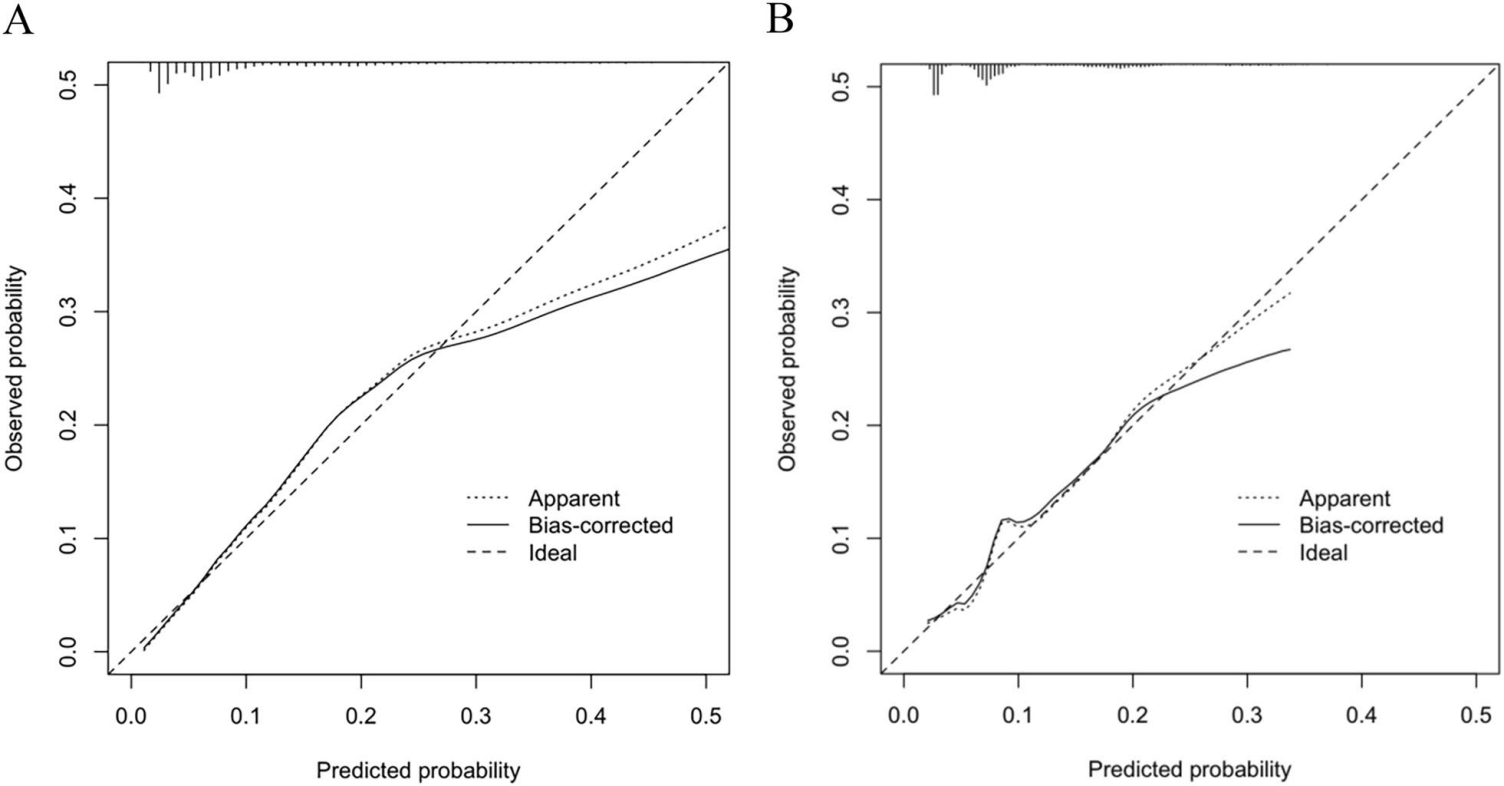
Calibration curves. **A** training set; **B** validation set

**Fig. 6:**
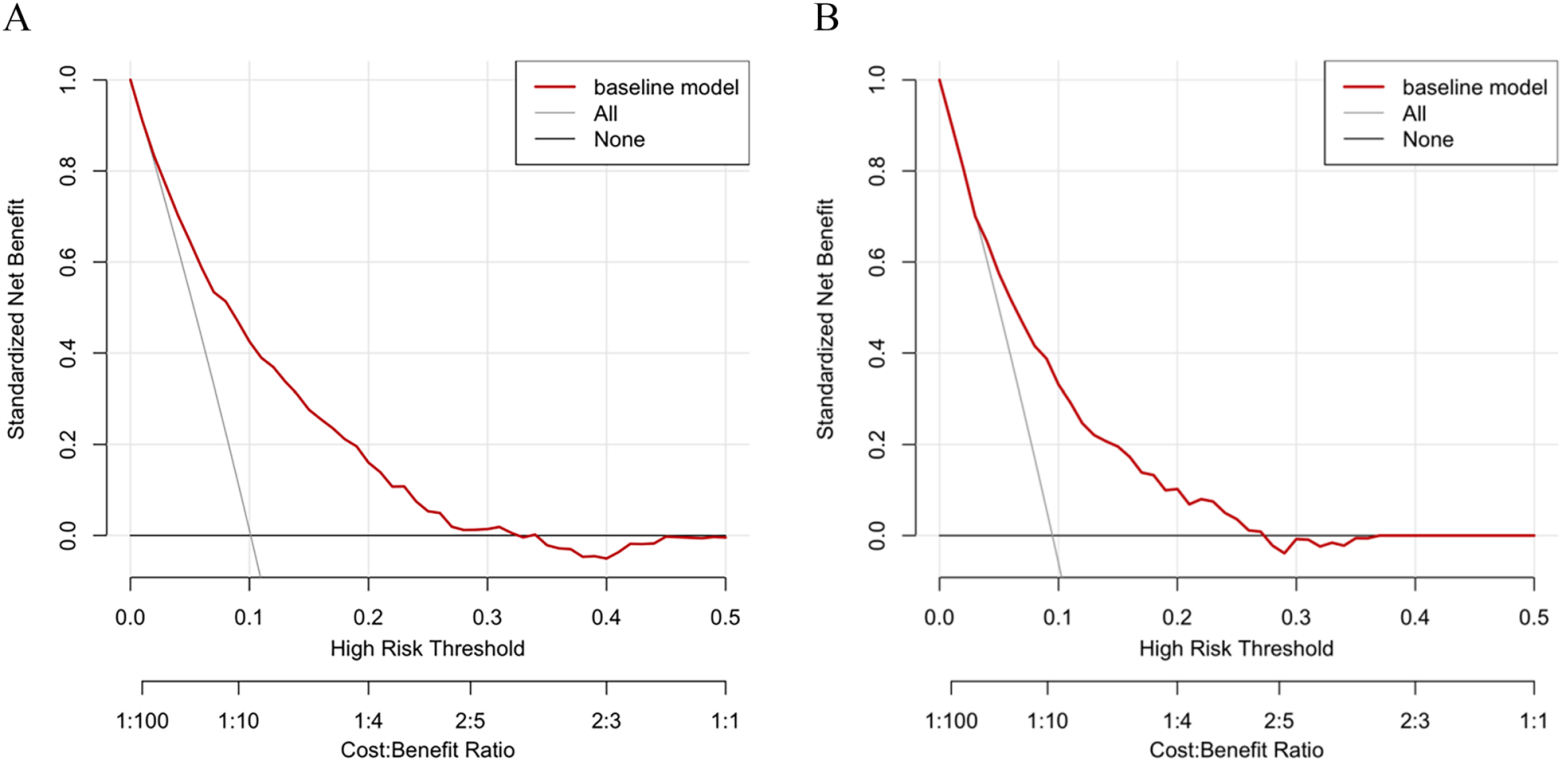
Decision curve analysis. **A** training set; **B** validation set

To visualize the prediction model constructed based on the results of multivariate Logistic regression analysis, a nomogram containing the 7 variables (Fig. 3) was developed, and the accuracy, calibration ability, and clinical utility of the prediction model were evaluated in the training set and the validation set. The C-index of the model in the training set and validation set was 0.77 (0.75,0.81) and 0.73 (0.75,0.81), respectively, and the AUC was 0.77 (0.74,0.80) and 0.73 (0.67,0.78), respectively. These results indicated that the prediction model has good discrimination power (Fig. 4). Additionally, the goodness-of-fit of our model was tested using the Hosmer–Lemeshow test, yielding a p-value of 0.57, suggesting a good fit of the model to the observed data. The calibration curves of the training set and the validation set had a good agreement with the diagonal (Fig. 5). The DCA curve, a measure of the clinical applicability of the predictive model, showed that the net benefit of the nomogram was higher than that of the "all treatment" or "no treatment" regimens (Fig. 6), suggesting that the model had favorable accuracy and predictive power for acute respiratory failure.

## Discussion

In this study, predictors were screened through LASSO logistic analysis, and a prediction model for the risk of acute respiratory failure in elderly patients with hip fracture was constructed based on 7 variables, including age, height, albumin, chloride, pneumonia, AKI, and heparin. The model showed good performance in both the training and validation sets, the calibration curve had a good match with the diagonal, and the DCA curve showed that the nomogram had more net benefit than the "all treatment" and "no treatment" regimens.

### Age was associated with respiratory failure after femoral fracture

Older people are more likely to have a hip fracture than younger people because they are more prone to osteoporosis. As they age, their balance declines and their risk of falls increases [23, 24]. In addition, the structure and function of the respiratory system of the elderly have changed, such as decreased lung compliance and reduced respiratory muscle strength, which affect respiratory function and oxygenation in the elderly [25]. The immune system of the elderly is also relatively weaker, which cause the elderly more susceptible to infection of respiratory pathogens [26]. It should be noted that hip fractures in the elderly often require prolonged bed rest, which can result in decreased respiratory muscle strength, increasing the risk of respiratory failure [9].

Surprisingly, our study results demonstrated that, within the population of older patients with hip fractures, those who are relatively younger exhibited a higher incidence of the disease. This phenomenon may be attributable to the following factors: ① disease base: If the total number of relatively younger old patients with hip fractures is small, but the count of patients with acute respiratory failure within this group is large, then the proportion of acute respiratory failure in this age group would be higher. Wang MT et al. found that the 10-year complication rate of hip fracture in young people was 26.50%, which was higher than that of the elderly (15.30%) [27]. ② Severity of disease: If a young person has a hip fracture, it may be due to a more serious accident, such as a high fall or a traffic accident, which may cause greater trauma to their body and increase the risk of acute respiratory failure [28].

### Height is associated with respiratory failure after a femoral fracture

There was a positive correlation between height and lung volume. If the height is shortened with age or disease, such as osteoporosis and scoliosis, lung volume would decrease, which would affect respiratory function [29, 30]. Moreover, there is a negative correlation between height and obesity. A shorter height was associated with a higher risk of obesity. Obesity can increase breathing work, lower respiratory compliance, and reduce functional residual capacity, making it a risk factor for respiratory failure [31]. Studies have shown that height may be associated with bone health, which in turn is influenced by nutritional status [32]. Loss of height with advancing age is often associated with degenerative changes in bone, such as osteoporosis, as well as compression of the intervertebral disc [33]. Optimization of nutrient intake is considered to be essential to combat the physiological aging of bone, able to slow this process by maintaining bone mineral density and the integrity of bone structure [34]. Ensuring adequate dietary intake of calcium and vitamin D is a key factor in slowing the rate of bone degradation associated with aging [35]. Malnutrition is associated with decreased immune function, delayed wound healing, and difficulty in rehabilitation, and it is a risk factor for complications after respiratory failure and hip fracture [36].

### Albumin levels are associated with the risk of respiratory failure after a femoral fracture

Hypoalbuminemia is a risk factor for respiratory failure because hypoalbuminemia can lead to higher pulmonary capillary permeability, increased pulmonary interstitial edema, and decreased oxygenation capacity, thereby increasing the risk of respiratory failure [37]. Hypoalbuminemia is also a risk factor for hip fracture because it can lead to decreased bone mass, damaged bone microstructure, and decreased bone strength, thereby increasing the risk of hip fracture [38]. Hypoalbuminemia may reflect a large surgical trauma, which is a risk factor for respiratory failure and complications after hip fracture because surgical trauma can lead to blood loss, infection, and hypoxia [39].

### Electrolyte chloride ions are associated with the risk of respiratory failure following a femoral fracture

Hypochloremia is a risk factor for complications after hip fracture, because hypochloremia leads to increased reabsorption of sodium by renal tubules, which increases sodium levels in the blood. In order to maintain sodium homeostasis, the body will increase urine volume, resulting in complications such as dehydration, hypotension, and shock [40]. Hypochloremia may reflect the presence of other chronic diseases because it can lead to renal insufficiency, excessive use of diuretics, vomiting, or diarrhea, which can reduce chloride levels in the blood. Chronic diseases are a risk factor for complications after respiratory failure and hip fracture, because chronic diseases can lead to systemic inflammatory response, organ dysfunction, and complex drug therapy [41].

### Pneumonia is associated with respiratory failure following a femoral fracture

Pneumonia is a common cause of respiratory failure because it can lead to ventilation/blood flow imbalance, diffusion disturbance, decreased lung compliance, and decreased respiratory muscle strength, resulting in hypoxemia and/or hypercapnia [42]. Pneumonia is a common postoperative complication, especially in patients undergoing hip replacement or internal fixation for hip fractures [43]. The incidence of pneumonia is related to factors such as operation time, age, complications, and preoperative status. At the same time, a hip fracture can also lead to bed rest, limited activity, and analgesic drug use, thus affecting respiratory function [44].

### AKI is associated with respiratory failure after femoral fracture

If patients develop AKI after a hip fracture, they may be at higher risk of developing respiratory failure. Postoperative acute renal injury will lead to deterioration of respiratory function, because it will lead to water retention, pulmonary edema, and decreased oxygenation capacity, thereby resulting in hypoxic respiratory failure [45]. Postoperative acute renal injury will also cause acidosis, potassium ion disorder, and calcium ion disorder, thus affecting neuromuscular function and respiratory drive, leading to hypercapnia respiratory failure [46].

### Heparin therapy is associated with respiratory failure after fetal fracture

Heparin is a commonly used postoperative prophylactic drug, which can prevent postoperative deep vein thrombosis by inhibiting thrombin activity [47]. Heparin therapy may be beneficial for respiratory failure because it can prevent and treat respiratory microcirculation disorders and pulmonary edema caused by pulmonary embolism, infection, and trauma [48].

Surprisingly, our results indicated that the incidence of the disease increased in the elderly who received heparin therapy, which may be related to the following factors: ① severity of the disease: Heparin is commonly used to prevent or treat thrombosis. Patients treated with heparin may be severely ill or have a higher risk of thrombosis, which makes them more susceptible to acute respiratory failure. ② Complications: The use of heparin may cause complications such as bleeding and heparin-induced thrombocytopenia [49]. These complications may increase the burden on the patient, thereby increasing the risk of acute respiratory failure. ③ Therapeutic response: Some patients may be resistant or intolerant to heparin [50].

## Generalizability and economic and clinical implications of the study

Considering the generalizability and economic implications of our study, it is crucial to note that the research was based on the MIMIC-IV database, primarily representing a large tertiary care center population in the USA. Therefore, the results might not directly apply to other types of hospitals or diverse healthcare systems. Nevertheless, the identified predictors in our model, such as age, height, albumin levels, are universally accepted and commonly measured factors in clinical practice, suggesting a broad potential application of our model.

Simultaneously, our model's economic implications should not be overlooked. The variables utilized in our model are routinely collected in clinical settings, thus requiring no additional cost for data collection. Moreover, by accurately identifying patients at high risk of respiratory failure, our model can guide timely interventions, which may lead to improved patient outcomes, shorter hospital stays, and reduced healthcare costs. Despite these advantages, further validation of this model in diverse patient populations and different clinical settings is still recommended to ensure its reliability and broad applicability.

## Limitations of the study

Nevertheless, our study had some limitations. ① Although the sample size was large, this was a single-center retrospective cross-sectional study with inevitable bias; ② while our model showed promising results with the current dataset, its applicability to diverse patient groups, especially across different regions and ethnicities, requires further validation; ③ hip fractures were not categorized by type, potentially introducing bias; ④ our model relied on available clinical variables from the database, omitting potentially relevant factors like genetics and lifestyle; ⑤ there was a lack of external validation, notably from domestic datasets. Prospective, multi-center studies will be pivotal in refining the model. Future iterations should expand the sample size, test across different populations, and incorporate broader influencing factors; ⑥ our choice to handle missing data with a 40% threshold, while preserving sample size, may introduce biases. Although our missing data were random, future studies should adopt stricter thresholds and enhanced data cleaning techniques; ⑦ the age inclusion criterion set in our study, at 55 years and above, diverges from the World Health Organization (WHO)'s definition of old age beginning at 60 years. A threshold of 55 years was selected to maintain a larger sample size of patients with acute respiratory failure, ensuring robustness in the analyses. However, this discrepancy might influence the generalizability of the findings to a broader elderly population defined by the WHO standards.

## Conclusion

In summary, the nomogram drawn in this study has good discrimination, calibration ability, and clinical utility, which can help clinicians better understand the risk of acute respiratory failure in elderly patients with hip fracture and then tailor medical intervention measures. The model can not only provide a reference for disease risk stratification, but also help to develop prognostic treatment strategies and follow-up strategies for survival.

## Data Availability

The raw data supporting the conclusions of this article will be made available by the authors, without undue reservation.

## Abbreviations

MIMIC: Medical information mart for intensive care
HIPAA: Health Insurance Portability and Accountability Act
PT: Prothrombin time
RDW: Erythrocyte hemoglobin distribution width
COPD: Chronic obstructive pulmonary disease
SAPSII: Simplified Acute Physiology Score II
K-S: Kolmogorov–Smirnov
OR: Odds ratio
CI: Confidence interval
HIT: Heparin-induced thrombocytopenia
ROC: Receiver operating characteristic
C-index: Concordance index
DCA: Decision curve analysis
LASSO: Least Absolute Shrinkage and Selection Operator
AKI: Acute kidney injury
AUC: Area under the curve
TRIPOD: Transparent Reporting of a multivariable prediction model for Individual Prognosis Or Diagnosis
ICD: International Classification of Disease
PaO_2_: Oxygen partial pressure
PaCO_2_: Carbon dioxide partial pressure
SOFA: Sequential Organ Failure Assessment
SQL: Structured query language
BMI: Body mass index
CK: Creatine kinase
CRP: C-reactive protein
ALT: Alanine aminotransferase
AST: Aspartate aminotransferase
Lambda.1se: Lambda.1se refers to the value of the regularization parameter lambda at which the cross-validated error is within one standard error of the minimum error observed in the regularization path of a LASSO model. This criterion is often used as a more conservative approach to selecting the model, aiming to improve model generalizability by trading off some bias for lower variance
